# Supplementary figures and images for: Beyond the ORF: Paralog-specific regulation of RPS7/eS7 mRNAs via 3’-UTRs and promoter sequences
Source: PLoS One. 2025 May 30;20(5):e0324525. doi: 10.1371/journal.pone.0324525 (PMC12124516; doi:10.1371/journal.pone.0324525)

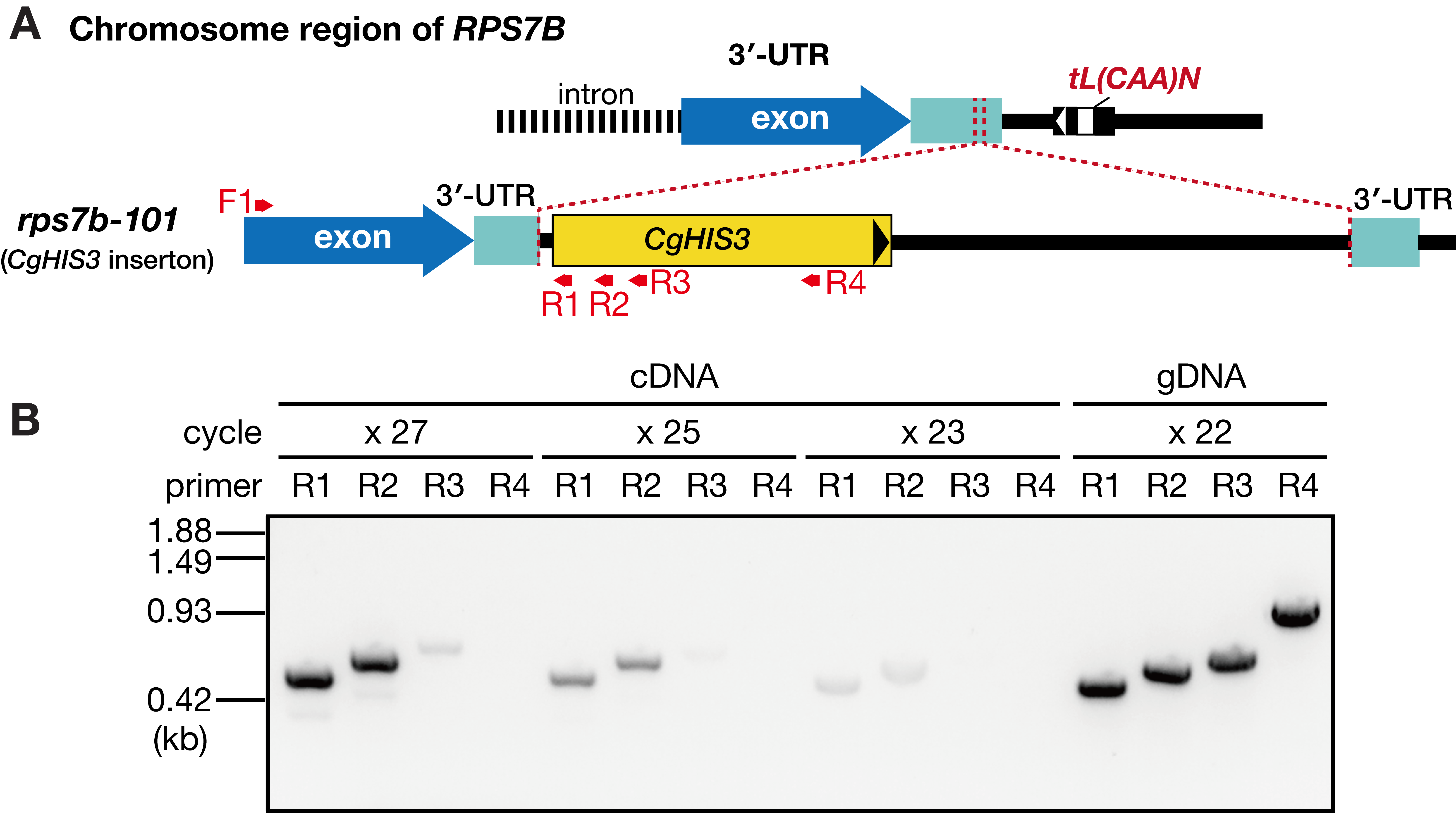

Supplement: S2 Fig — (A) Three prime parts of the rps7b-101 gene and positions of primers used in the PCR (small red arrows) were schematically shown. (B) The 3’ parts of the rps7b-101 were amplified from SHSC0388 (rps7b-101) cDNA and were analyzed as in S1 Fig. Primers used in the PCR are shown on the top of the gel image, and correspond to those in (A). The rightmost four lanes are PCR products with genomic DNA from SHSC0388 as a template, and are used to confirm ability of primers. (TIF) [file pone.0324525.s002.tif]
