# Supplementary material for: Beyond the ORF: Paralog-specific regulation of RPS7/eS7 mRNAs via 3’-UTRs and promoter sequences
Source: PLoS One. 2025 May 30;20(5):e0324525. doi: 10.1371/journal.pone.0324525 (PMC12124516; doi:10.1371/journal.pone.0324525)

Fig 1B

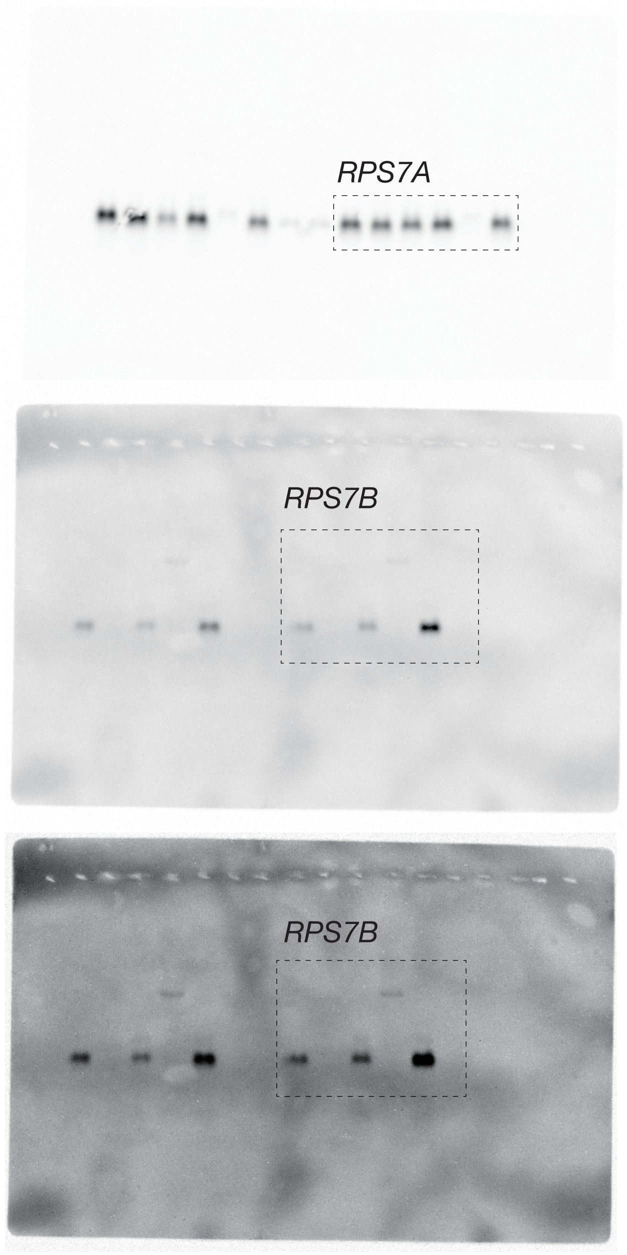

Fig 2C

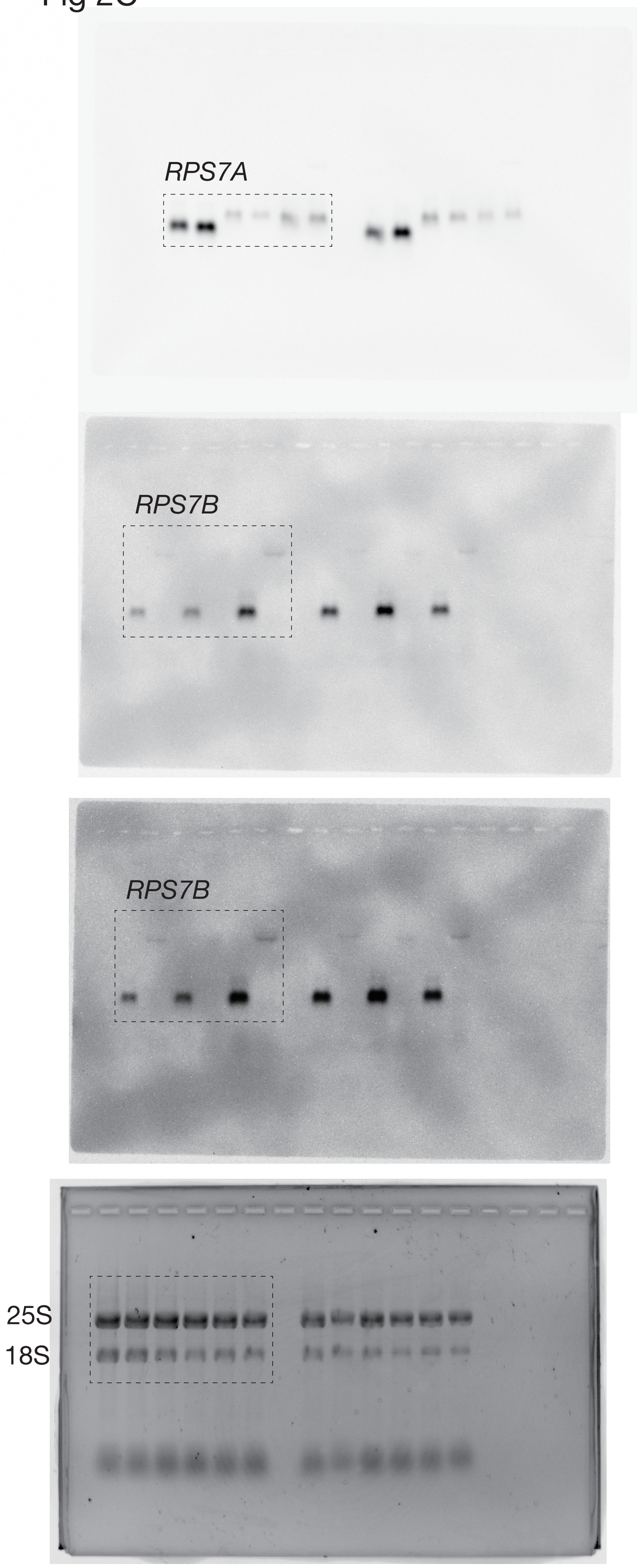

Fig 1C

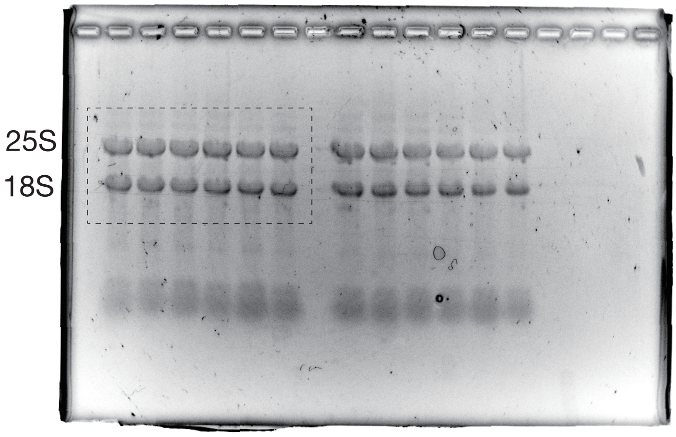

Fig 2D

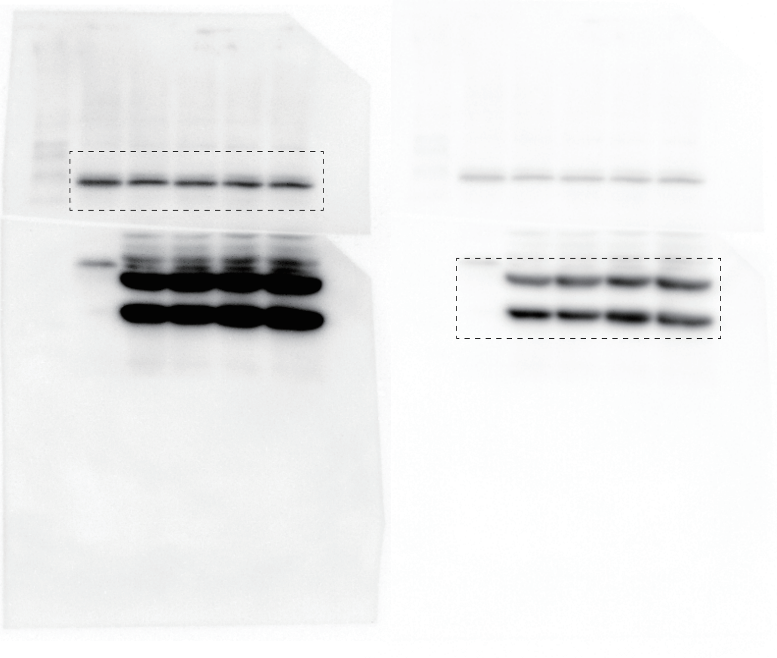

Fig 3C

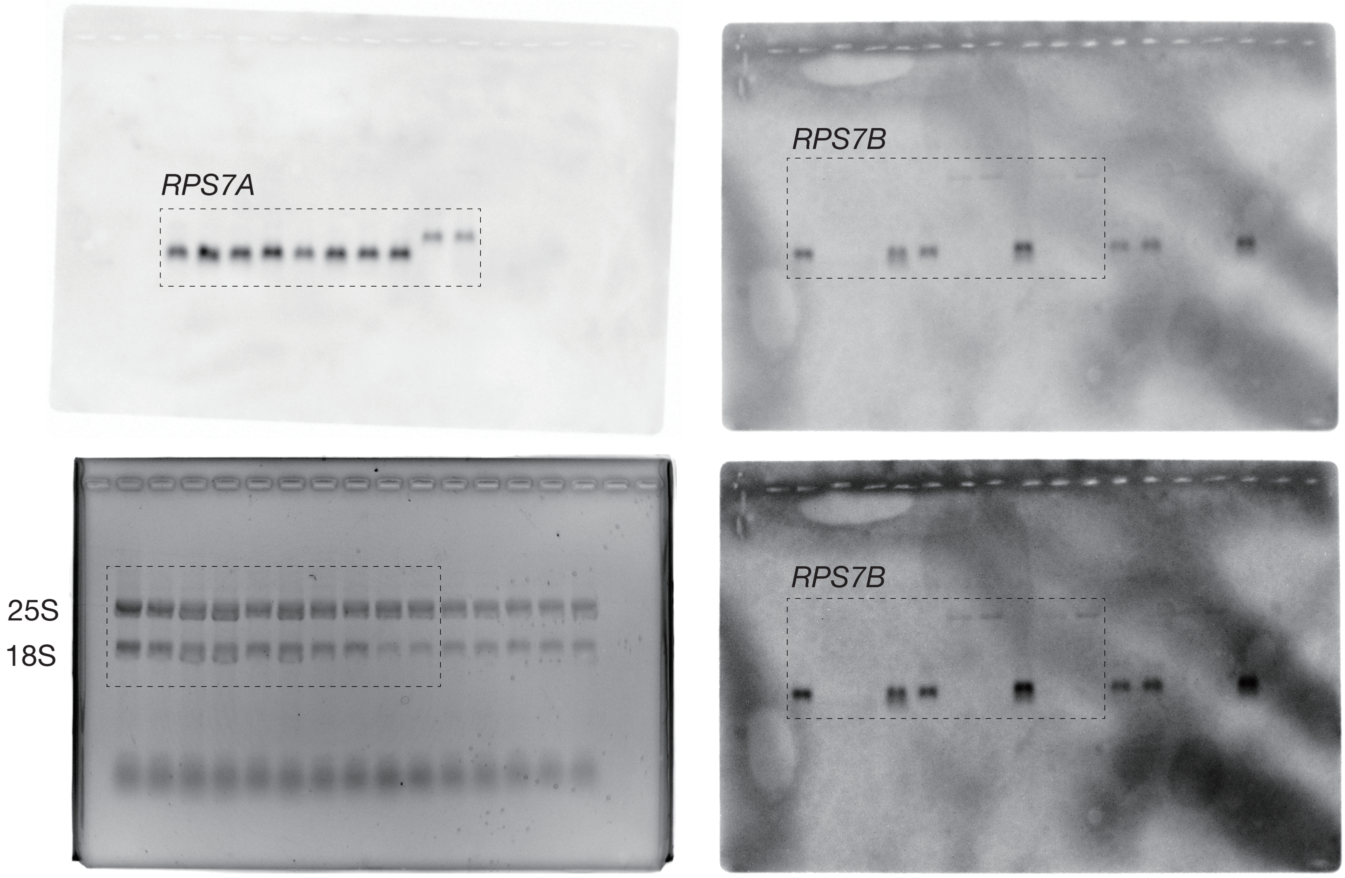

Fig 4C

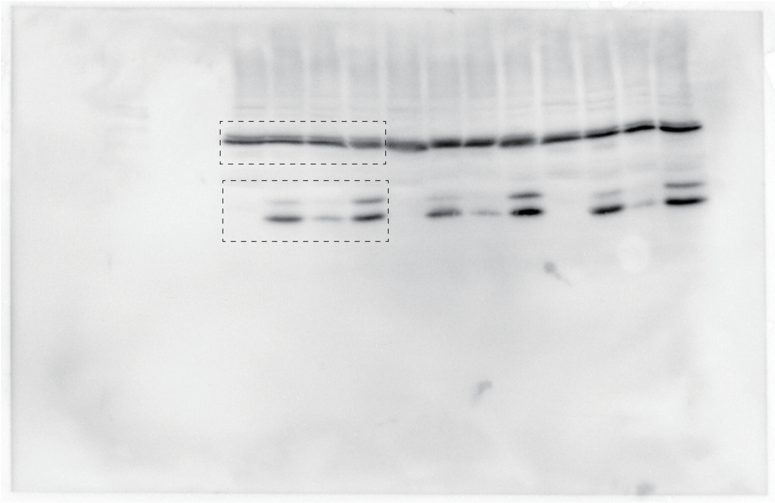

Fig 4D

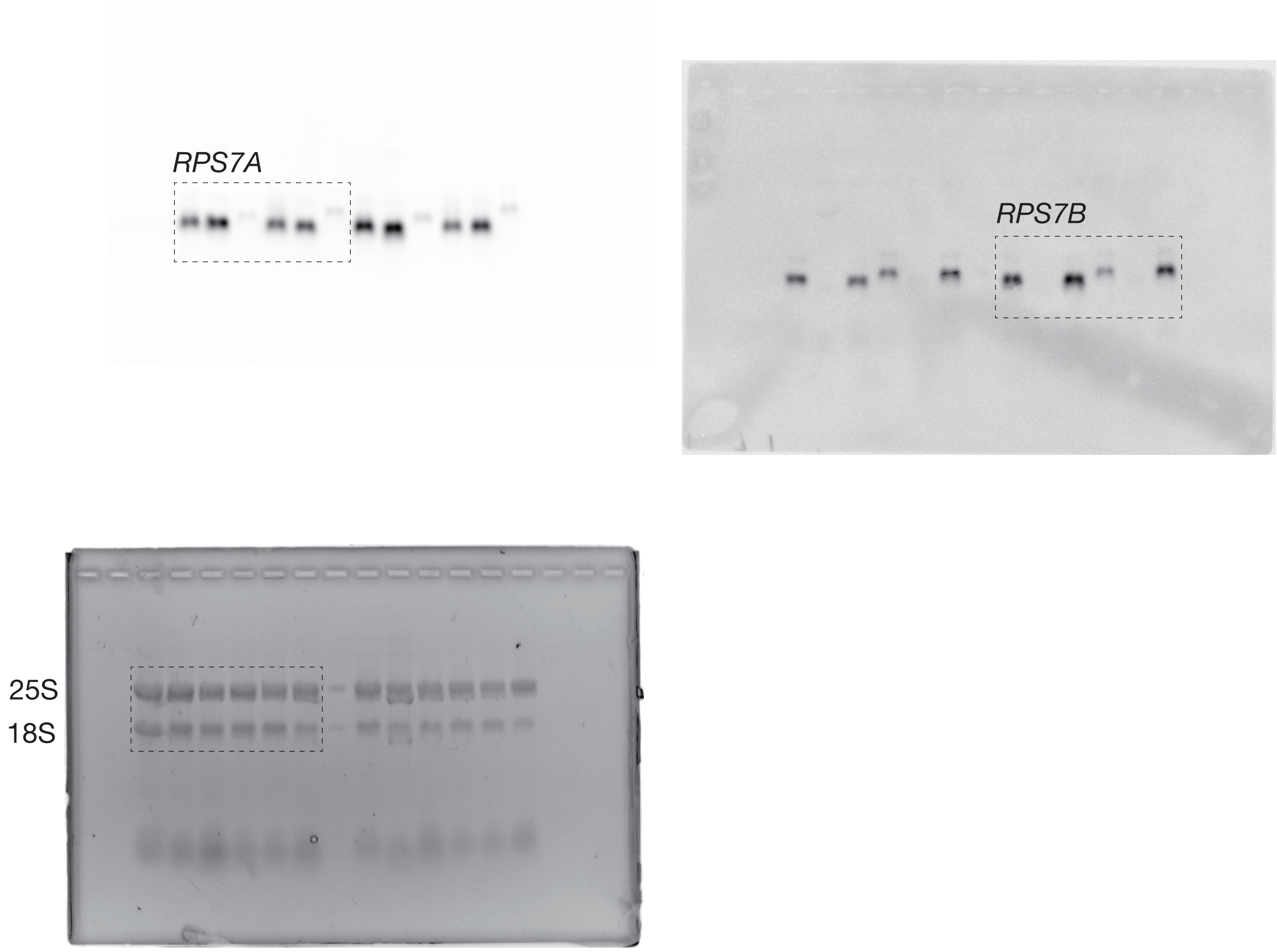

Fig 5B

WT

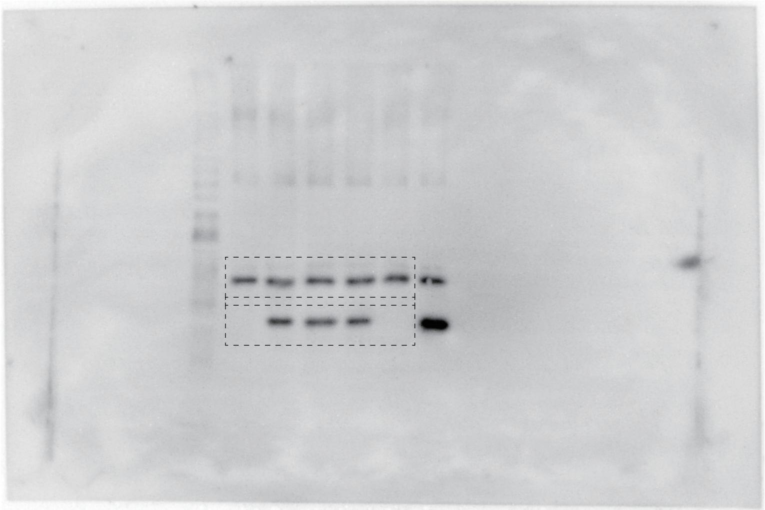

*rps7a*Δ

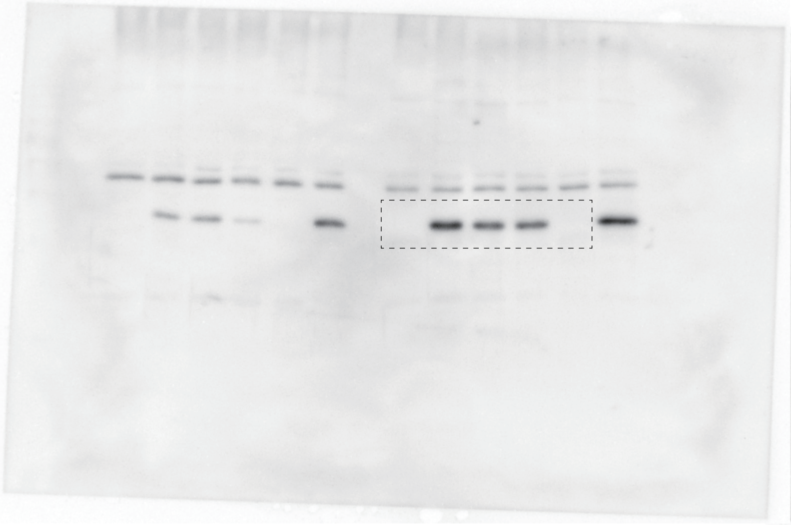

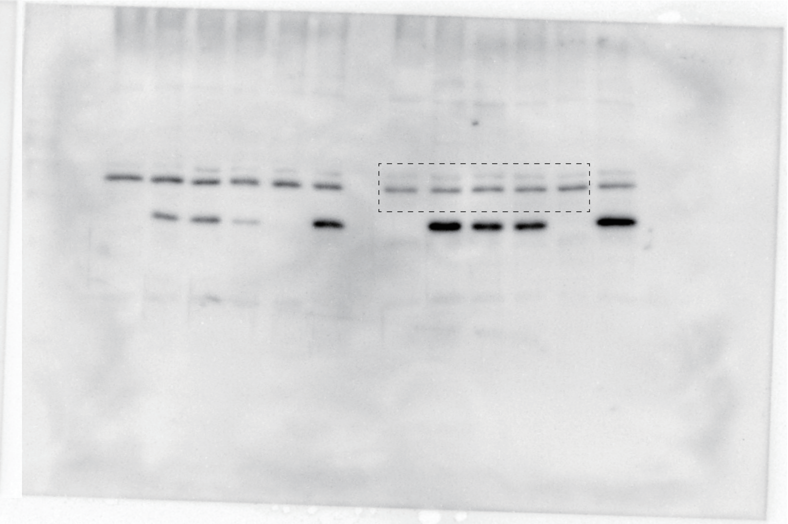

*rps7b*Δ

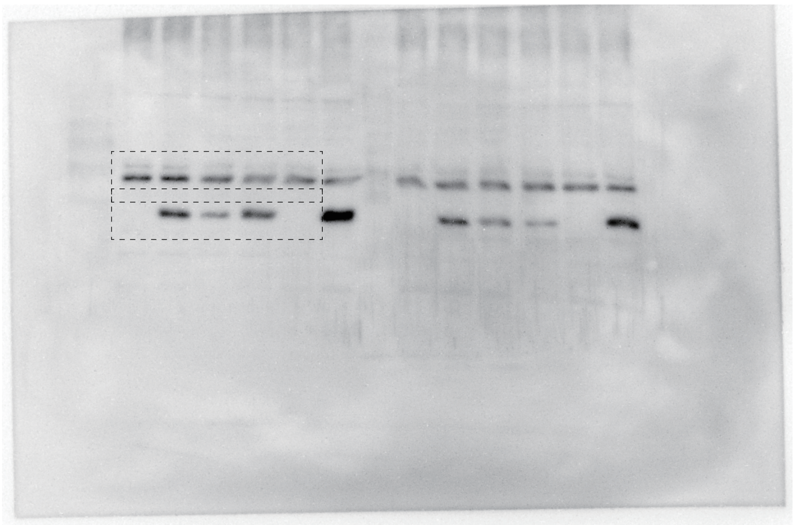

Fig 5C

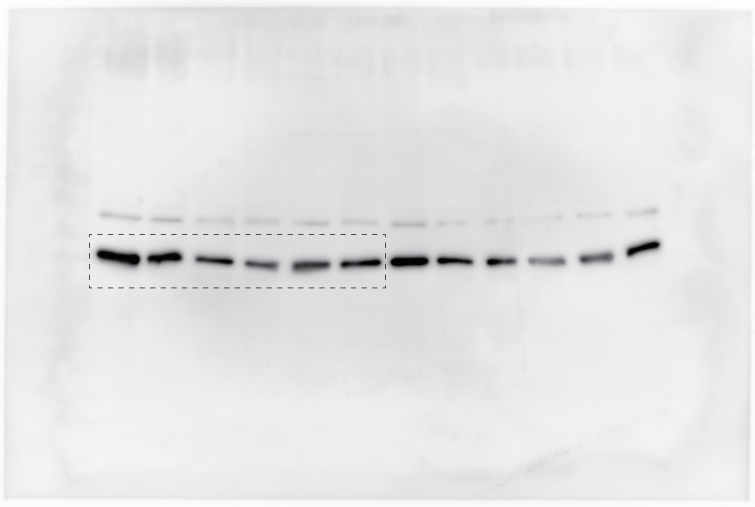

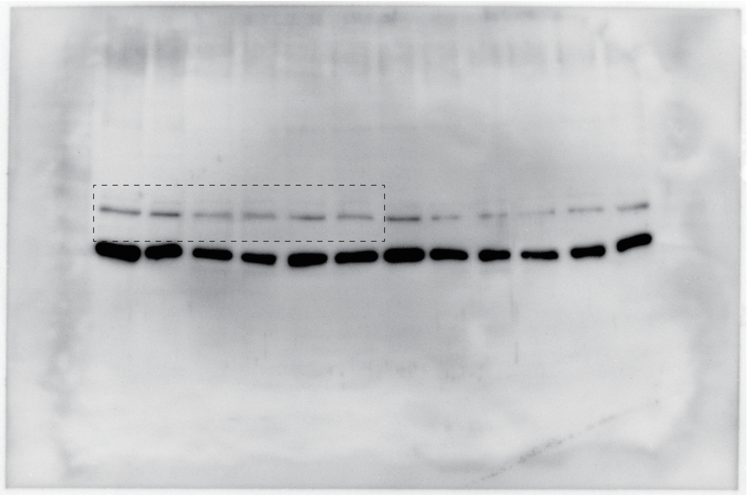

Fig 5D

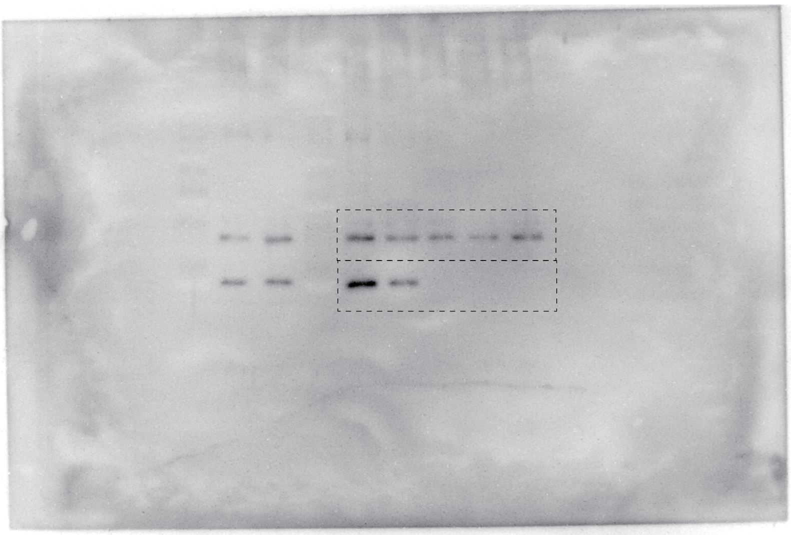

S1 Fig. panel B

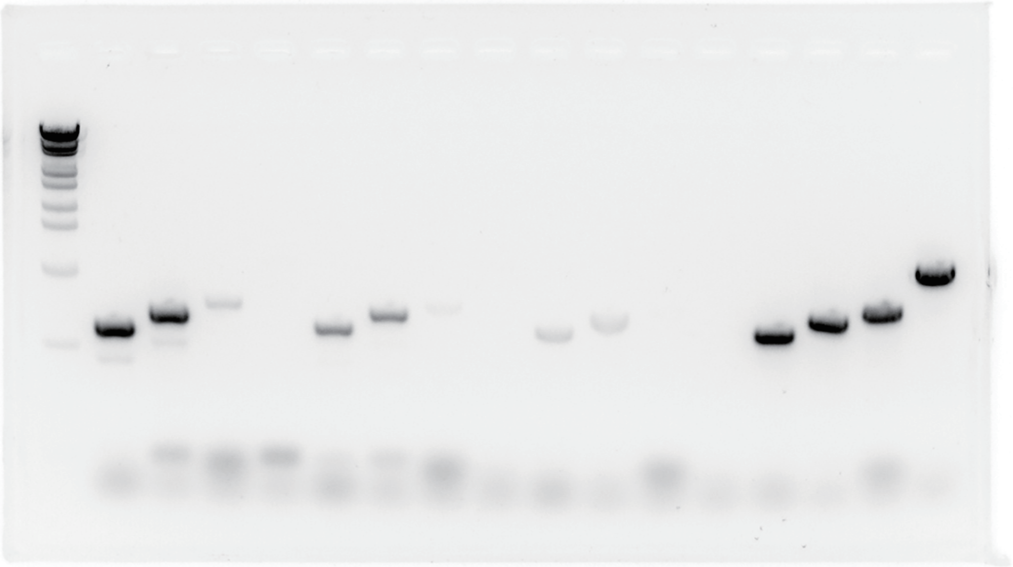

S2 Fig. panel B

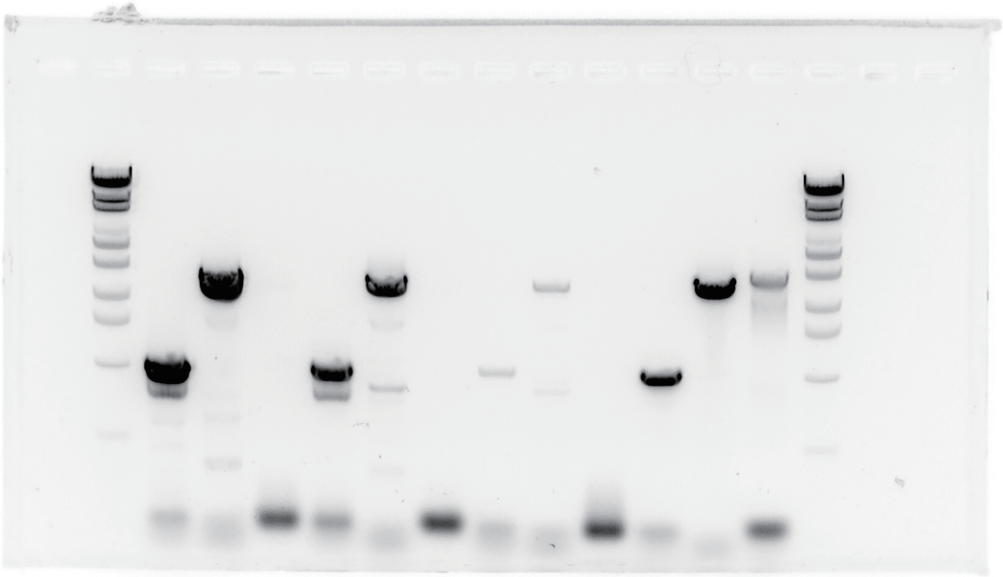

S3 Fig. panel B, RPS7A unspliced

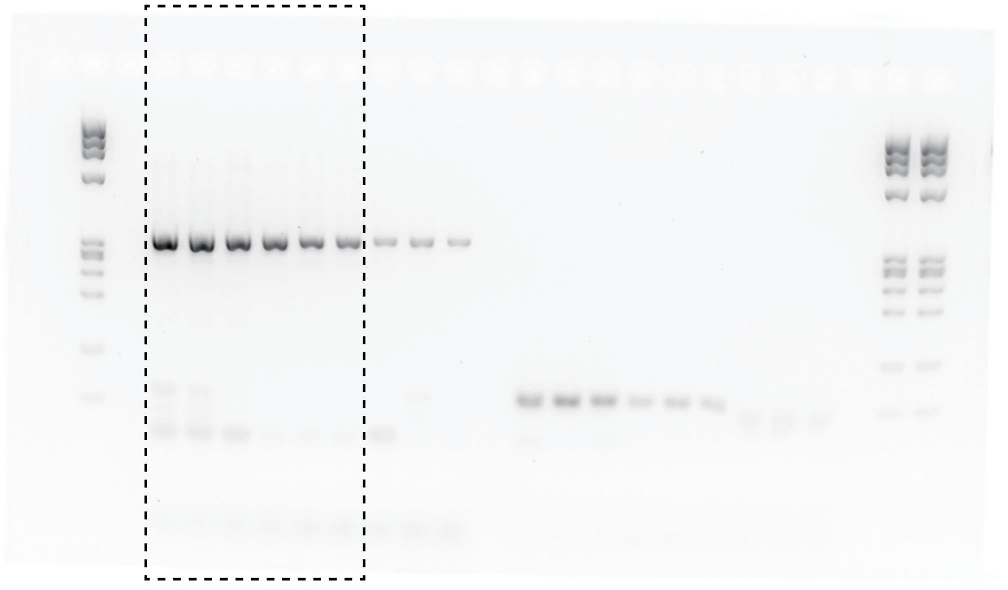

S3 Fig. panel B, RPS7A spliced

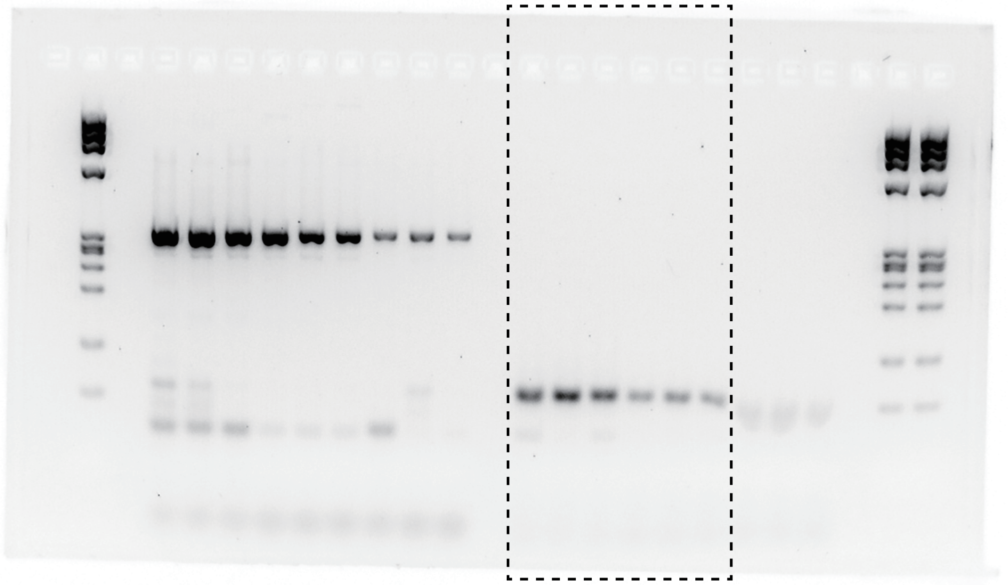

S3 Fig. panel B, RPS7B unspliced

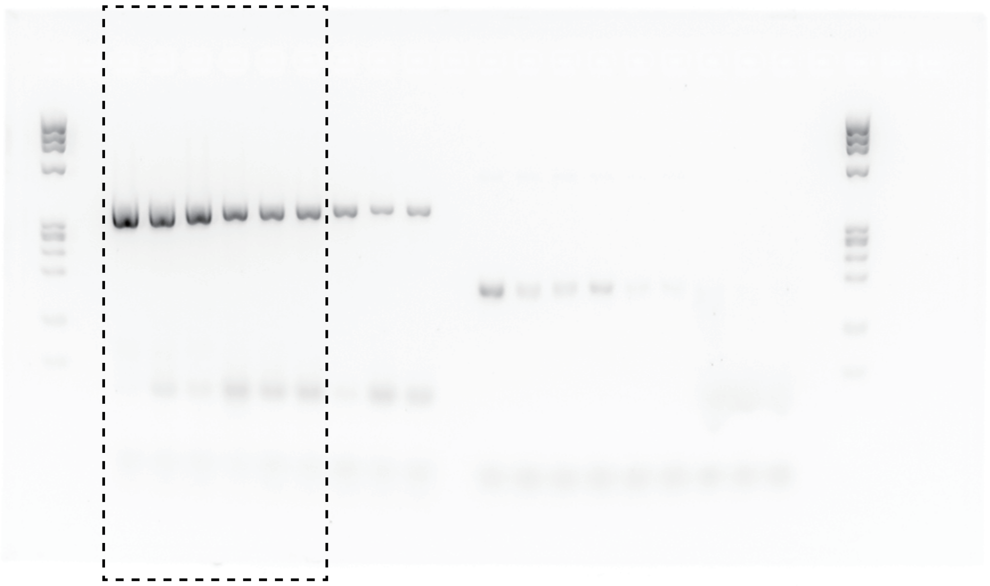

S3 Fig. panel B, RPS7B spliced

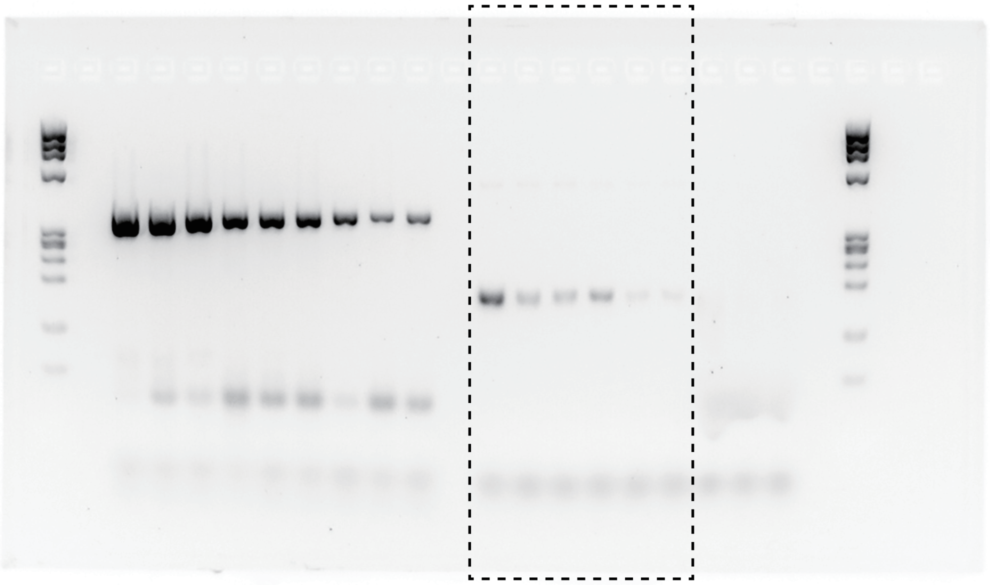

Supplement: S2 File — (PDF) [file pone.0324525.s005.pdf]
