# Supplementary material for: Beyond the ORF: Paralog-specific regulation of RPS7/eS7 mRNAs via 3’-UTRs and promoter sequences
Source: PLoS One. 2025 May 30;20(5):e0324525. doi: 10.1371/journal.pone.0324525 (PMC12124516; doi:10.1371/journal.pone.0324525)
Supplement: Attachment — Submitted filename: Response to Reviewers.pdf [file pone.0324525.s007.pdf]

Important note:
Please refer to the page and line numbers indicated in "**Revised Manuscript with Track Changes.pdf**" for review. **This file includes track changes and highlights in yellow the sections pointed out by the reviewers.**

Point-to-point answers
5. Review Comments to the Author

Reviewer #1: The work of Hayashi and Yoshihisa is a demonstration of the use of scientific reasoning to investigate the molecular mechanisms of life from a serendipitous finding. The work contains many results, with varying relevance. Some of them serve to interpret the phenotype of the spontaneous mutant, which has little biological relevance, and others focus on the study of a new mechanism of cross-control between paralogous copies of a yeast ribosomal protein.

As you pointed out, we originally interested in tRNA biology and began the manuscript with our unexpected (artificial) mutant and the observation made from this mutant. While the biological relevance of our mutant is limited, considering that tRNA gene regions are often targeted by transposon (Guimarães, *et al.*, 2021, Front. Microbiol., 12, 634004, doi: 10.3389/fmicb.2021.634004), such genomic changes altering the 3'-UTR of *RPS7B* could occur during evolution (and were probably eliminated by natural selection). Therefore, we believe our serendipitous finding holds significance, at some level, within the context of the manuscript. At this point, we added this to the discussion. Please refer to the following.

Page 22, lines 426-431:
Our serendipitous finding began with an unexpected genomic change near a tRNA gene during construction of the *tl(caa)Δint* strain. Although our case is completely artificial, insertion are often introduced at genomic fragile sites by transposons [34]. Indeed, tRNA loci exhibit mutation rates approximately 7 to 10 times greater than the genome-wide average [35]. Thus, genomic changes or mutations similar to our mutant might arise under natural conditions during evolution.

1) *On page 13, line 194, the mRNA of the CaHIS3 insertion version in Fig. 2C is longer than wt. This should be mentioned in the text.*

Thank you for your advice. We revised the sentence, including the corresponding part as below.

Page 13, lines 231-233:

The relative levels of *RPS7A-HA::CgHIS3* mRNA, which is longer than wild-type *RPS7A*, were approximately 70-80% lower than those of wild-type *RPS7A* across all backgrounds (Fig 2C).

2) *On page 13, line 199, the Rps7A protein level in wt is not shown. This is because wt is not HA-tagged, but it would be interesting to have a relative quantification of Rps7A levels in Rps7A-CgHIS3 with respect to those in wt.*

We agree with your idea. Unfortunately, we don't have an antibody against Rps7A, which makes experimentation difficult. Based on the mRNA levels, Rps7A from *RPS7A::CgHIS3* may be reduced compared to that from wild-type *RPS7A*.

3) *Page 15, line 228. There are two ... at the end of the line.*

Thank you for pointing out. We deleted one "." Please refer to page 16, line 280.

4) Page 17, line 268. "we deleted this sequence...". It is important to know which sequence was deleted. Since there is a putative 5 bp overlap between the Rap1 and Fhl1 binding sites, if the entire sequence is deleted the consequence would be a lack of both binding sites. If only part of it is removed, Rap1 (or Fhl1) may still bind to it. According to line 272 on page 18 both sites were removed but this conflicts with other conclusions given later. See point #5 below.

We deleted the entire sequence covering the Rap1p and Fhl1p binding sites, thus both binding sites. We revised the sentence to clarify this point, and add a graphical representation of mutated sequences in Fig 5A.

Page 18, lines 339-340:

We deleted this entire sequence covering the Rap1p and Fhl1p binding sites to make a mutated version of the *RPS7A* promoter (Fig 5A, *rps7a-mut*).

Pages 41–42, lines 860-869

The right panel shows enlarged sequences of the dotted line areas from the left panel. The numbers represent the upstream region relative to the transcription start site. The simultaneous binding sites for Rap1p (*RPS7A*: 5'-ATGTTTGGGT-3'; *RPS7B*: 5'-AGGTACGGAT-3') and Fhl1p (*RPS7A*: 5'-ACCCAAACAT-3'; *RPS7B*: 5'-ATCCGTACCT-3') are highlighted in red,

while the Fhl1p binding site alone in *RPS7B* (5'-ATCCGTACAT-3') is shown in orange.

5) Line 272 states that (Fig. 5B) the lack of both Rap1/Fhl1 binding sites is not affecting Rps7A expression (as demonstrated by the GFP reporter). This is quite surprising because these factors are described as very important for RP gene expression in many previous papers. Here it appears that in the RPS7A gene they are only required for negative feedback of the Rps7 protein on RPS7A transcription. Although the question is discussed below (pages 23-24) it is quite surprising and as no potential mechanism is suggested it leaves the question too unresolved. Is the Rps7 protein interacting with Rap1 (or Fhl1, or another factor) to prevent binding? If so, the experiment shown below (fhl1 mutant) will demonstrate that Fhl1 is NOT the target of Rps7A. As for the function of Rap1, since Rap1 is essential a ts mutant or Auxin-degron can be used to test the function of Rap1 in Rps7A transcription. Alternatively the rap1delta-sil mutant has been used in the context of RP gene expression where it affects the RNA pol II transition between initiation and elongation (doi:10.1371/journal.pgen.1000614).

We appreciated your variable comment and discussion. We aim to express ideas with confidence only when supported by robust evidence. Currently, we unfortunately lack comprehensive data on the regulatory mechanism governing the *RPS7A* gene, which is primary involved in the negative feedback of Rps7 protein on *RPS7A* transcription. Nevertheless, as discussed in pages 25-26, lines 509–524, we need to suppose Fhl1p's function independent of the Fhl1p site in the *RPS7A* promoter region to explain the effect of the *rps7a-mut fhl1*Δ double mutant, and this is the utmost mechanistic suggestion we can make at this stage. While we refrain from further speculating on potential interactions with other regulators in the present manuscript, we included the following description in discussion.

Page 27, lines 544-549:
Moreover, Rps7Ap may directly interact with a transcriptional regulator to achieve *RPS7A*-specific feedback regulation, as known by the general autoregulatory mechanism of RP production [3]. At least, Fhl1p is not the target of Rps7A (Fig 5), indicating that Rap1p is the prime target identified so far. While Rap1p is essential, the use of a *ts*-mutant [47,48] or Auxin-degron system, and the *rap1*Δ*sil* mutant [49] offers valuable tool for further investigation.

6) page 24 line 373. RPS7 is RPS7B?

We intended for "*RPS7*" to refer to both *RPS7A* and *RPS7B*. To clarify, we revised the term to "*RPS7A/B*". Please refer to page 26, line 516.

7) In Discussion (page 24) the authors describe the existence of other TFs that can be activating Rps7A promoter, such as Ifh1 or Hmo1. I think that Sfp1 should be included because it another well-known transactivator of many RP genes.

Thank you for your suggestion. We revised the manuscript to include Sfp1 as one of the other potential TFs.

Page 26, lines 517-521:
This suggests that the Fhl1p site in the *RPS7A* promoter is dispensable for Fhl1p function, as Fhl1p can still be recruited to the *RPS7A* promoter via interactions with other transcription factors, such as Rap1p (a pioneer transcription factor [42]) and Sfp1p (a transcription factor requited by Rap1p [40]), and/or Hmo1p (an HMG family chromatin interactor [43]), which associated with the *RPS7A* promoter region by themselves [40,43,44].

Reviewer #2: The authors focused on a very interesting topic: what does it happen with paralogous genes? how redundant/specific are they? They have focused on a pair of ribosomal proteins (RPS7A and RPS7B). The main problem of the paper is that the main aim of the study, the specific objectives and the hypotheses are never clearly stated upfront. Therefore, the reader does not know if the described experiments make sense in order to achieve that aim and answer those research questions.

Thank you for your valuable feedback. Based on your comments, we revised the entire manuscript, with particular focus on the results section, to provide the objectives and/or hypotheses presented more clearly at the outset. Please refer the corresponding parts as follows.

Page 13, lines 219-223:
Paralogous RP gene expression is regulated by multiple layers of transcriptional and post-transcriptional levels [3]. In trypanosomatids, recent findings show that the translation of RP genes can be regulated by RNA-binding proteins interacting with their 5'- or 3'-UTRs [27], contributing to paralog-specific expression [28]. Similarly, in yeast, UTRs may serve as regulatory elements for RP paralog expression, potentially playing a more significant role than previously anticipated.

Page 14, lines 242-243:
Collectively, the 3'-UTR of *RPS7A* seems to have some *cis* element(s) to enhance the basal

transcription and/or stability of *RPS7A* mRNA.

Page 14, lines 248-251:
The presence or absence of an intron in a tRNA gene influences its ability to function as an insulator, preventing the spread of silenced chromatin and thereby altering the silencing status of nearby pol II genes [29–31]. Thus, it is possible that the tRNA intron in *tL(CAA)N* additionally affect *RPS7B* expression in the *tl(caa)Δint** strain. To verify whether…

Page 16, lines 280-281:
And, the tRNA intron had no effect on *RPS7B* expression with the long 3'-UTR.

Page 16, lines 294-296
According to Ghulam *et al.* [7], Rps7p expression is regulated by both transcriptional and translational levels. To assess the transcriptional effect of *RPS7B*, we first performed semi-quantitative RT-PCR.

Page 17, lines 306-308:
We then examined whether the *tl(caa)Δint** strain impaired *RPS7B* mRNA expression post-transcriptionally, hypothesizing that mRNA abundance directly reflects protein levels due to aberrant 3'-UTR.

Page 18, lines 327-334:
In the traditional view, regulation of RPs often occurs at protein level or in response to the amount of assembled ribosomes, with feedback process frequently leading to transcriptional repression of RP genes [32]. Thus, *RPS7* paralog-specific feedback response would initiate at transcriptional level, and in this process, the upstream and/or downstream regions of the ORF may provide clues to understanding the *RPS7*-specific gene regulation mechanism *in vivo*. To explore difference of transcriptional features between *RPS7A* and *RPS7B* genes, we first focused on the upstream region and searched for known *cis* elements located in the promoter region of *RPS7A* and *RPS7B* on Saccharomyces Genome Database.

Page 20, lines 380-385
Collectively, the upstream region, particularly the Fhl1p and/or Rap1p binding sites, play a crucial role in *RPS7* paralog specific response.

Secondly, to evaluate the impact of the downstream region on their paralog-specific expressions, we additionally generated reporter genes in which constructs share a common

ADH1 promoter and *GFP-FLAG-HIS3* gene, with the only difference being the 3'-UTRs and terminators (*RPS7A* or *RPS7B*), in contrast to the upstream region.

Page 20, lines 390-392

Since Fhl1p and/or Rap1p binding sites are crucial for *RPS7*-paralog specific response (Fig 5B), we generated an *fhl1*Δ strain and repeated the reporter assay to analyze whether Fhl1p acts on *RPS7* paralog expression.

Page 21, lines 414-416

Thus, the different contributions of Fhl1p (and/or Rap1p) to the *RPS7*-paralog promoter regions are key determinants of *RPS7*-paralog expression, which is influenced by the levels of Rps7Ap (or total Rps7p).

In my opinion, some of the presented experiments seem irrelevant in order to understand the relationship between RPS7A and RPS7B. For example, the authors constructed a strain with an artificially long 3'UTR in RPS7B. Then, they demonstrate that NMD controls the expression of that modified RPS7B gene. However, I wonder why this experiment is relevant if this never occurs in natural/biotechnological strains. WT RPS7B has a normal 3'UTR which is probably not targeted by NMD. Therefore, the implications of these results are totally obscure to me.

Thank you for your opinion. We originally interested in tRNA biology and began the manuscript with our unexpected (artificial) mutant and the observation made from this mutant. While the biological relevance of our mutant is limited, considering that tRNA gene regions are often targeted by transposon (Guimarães, *et al*., 2021, Front. Microbiol., 12, 634004, doi: 10.3389/fmicb.2021.634004), such genomic changes altering the 3'-UTR of *RPS7B* could occur during evolution (and were probably eliminated by natural selection). Therefore, we believe our serendipitous finding holds significance, at some level, within the context of the manuscript. At this point, we added this to the discussion. Please refer to the following.

Page 22, lines 426-431:

Our serendipitous finding began with an unexpected genomic change near a tRNA gene during construction of the *tl(caa)*Δ*int* strain. Although our case is completely artificial, insertion are often introduced at genomic fragile sites by transposons [34]. Indeed, tRNA loci exhibit mutation rates approximately 7 to 10 times greater than the genome-wide average [35]. Thus, genomic changes or mutations similar to our mutant might arise under natural conditions during evolution.

Moreover, other comparisons do not seem to be absolutely fair. In my opinion, it is very interesting to know how the amount of mRNA and transcript of one of the paralogs is affected when the other paralog is knocked down or overexpressed. Here, the authors have looked at the effect of the RPS7A deletion on RPS7B. They have also looked at the effect of the RPS7B deletion on RPS7A. However, most of the quantitative analyses on RPS7A where done with the long-UTR RPS7B gene. In this experiment, the transcription of RPS7B is not prevented, but the mRNA is mostly degraded and hence never translated. The reciprocal experiment (RPS7A targeted by NMD) is never done. In order to rule out symmetrical effects, the analyses need to comprehensive.

We understand your points, and we agree that further comprehensive analyses are needed to rule out these symmetrical effects. As mentioned previously, the story presented in this manuscript began with our spontaneous mutant, which has *RPS7B* gene with the long 3'-UTR. This led us to focus more specifically on the location where the *RPS7B* gene derivatives are expressed. Further, according to the genomic context, there are no tRNA genes or fragile sites around *RPS7A*, suggesting that natural insertion would not occur. Therefore, we did not investigate the effect of a 3'-UTR insertion in the *RPS7A* 3'-UTR. We have added these descriptions including the limitations of our experiment.

Pages 24-25, lines 483-493:
We did not investigate the effect of a 3'-UTR insertion in the *RPS7A* 3'-UTR, specifically a 2.3-kb insertion of *loxP::CmR::hisG::loxP*, as seen in *rps7b-102*. Given the genomic context, there are no tRNA genes or fragile sites around *RPS7A*, suggesting that natural insertion would not occur. However, more comprehensive studies are needed to examine the impact of 3'-UTR insertions in *RPS7A* to better understand the overall imbalance in regulation between paralogous genes.

Finally, I am not entirely satisfied by the way that the statistical analyses are performed, reported and interpreted.
1) I did not see information on the number of replicates used in each experiment; n must be reported.

Thank you for pointing out. We described the number of replicates (n) for each experiment in the main text or in the figure legends.

2) t-test is used in most analyses, but this is incorrect because many of those involve multiple comparisons. If all comparisons are of interest, the authors should use ANOVA plus a post hoc test (e.g. Tukey's test). If only comparisons to a single control are of interest, Dunnet's test should be used instead. If not all the comparisons are of interest and there is no common control, the authors should correct the p-values of the t-test for multiple testing (because they are interested in any significant difference, their type-I error is above 0.05).

Thank you for your comment. We revised multiple comparisons approach from a *t*-test to pairwise comparison with Benjamini-Hochberg False Discovery Rate (FDR) method. The revised analyses were done in Fig 4C, 5B and 5C. The following descriptions for statistical analyses are revised.

Page 17, lines 313–314, and line 315, and Page 41, lines 849–850 for Fig. 4C.
Page 19, lines 349–350, lines 351–352, line 356, lines 357–358, line 362, Page 20, lines 378–379, Page 42, lines 874–875 for Fig 5B.
Page 42, lines 880–881 for Fig 5C.

According the re-analysis, the conclusion of the result in Fig 5B was changed, therefore we revised the description as fellows.

Page 20, lines 377-379:
While disruption of the *cis* element for Fhl1p and Rap1p in the *RPS7A* promoter seemed to reduce reporter expression, this was not statistically significant (Fig 5B; BH within the same *RPS7* genetic background, $p > 0.05$).

3) the authors disregarded an interesting result because it does not agree with their main conclusion (RPS7B expression depends on RPS7A expression, but RPS7A expression is not affected by RPS7B). When describing Figure 1B, the authors state "rps7bΔ affected RPS7A expression only slightly (rps7bΔ/WT, 0.70±0.13; by Student's t test, p =0.0066)" in lines 177-178. I totally disagree with this statement. First, the difference is either statistically significant or not; in this case, it clearly is (p-value < 0.01). Slightly is not a statistical term. Second, RPS7A expression is clearly depleted in the rps7bΔ strain; it is just 70% of the expression in the WT strain. A 30% depletion is not a slight change. This result should be taken into account when interpreting the rest of analyses.

We appreciate your criticism. To more precisely interpret and describe the result, we changed

the expression as follows.

Page 12, lines 200-202:
We also tested effect of *rps7b*Δ and found that the deletion affected *RPS7A* expression marginally (*rps7b*Δ/WT, 0.69±0.13; by Student's *t*-test, p = 0.0066; n=3).

For all these reasons, I have to recommend that the paper is rejected. I would suggest to the authors that they decided on what is the main aim of their research and then they did a complete rewriting of the paper focusing on achieving that aim.

Reviewer #3: Hayashi and Youshihisa present their results from investigating the regulation of the yeast RPS7A and B paralogs. Because the yeast genome resulted from a whole-genome duplication event, many proteins are duplicated (termed ohnologs). This includes many ribosomal proteins. Previous work has reported that the alternative copies of ribosomal proteins present in the yeast genome have different regulation and, potentially, somewhat different functions. In this manuscript, the authors report similarities and differences in the regulation of the expression for ohnologs RPS7A and RPS7B. In particular, they report that the short 3' UTR of both genes is important for RNA stability, and that the transcriptional regulation of both RPS7A and 7B respond to a decrease in RPS7A expression, suggesting a feedback loop. The authors also describe some differences in the functions of transcription factor binding sites upstream of the two genes. Overall, this is a well performed study that is interesting to the gene regulation and ribosome research communities. I have mostly minor comments that should be addressed:

1. It has been reported that deletions of ribosomal proteins can cause yeast to become aneuploid as a response to loss of the RP copy (see PMID: 30503772 and PMID: 22377630). To elaborate, segments or entire chromosomes carrying the RP copy that is still present will duplicate, as cells that have the "normal" number of copies are more fit than those that lose one copy (even with the aneuploidy). The authors should note this in the introduction and discussion, as it is relevant to their observation that RPS7A deletion leads to an increase in RPS7B mRNA. It is possible that these increases could be due, at least in part, to amplification of the remaining RPS7 DNA copy.

We appreciated your thoughtful opinion. According to your comment, we added the description in introduction and discussion parts as follows.

Introduction; page 3, lines 47-49:
Further, deletion of RP genes can lead to aneuploidy in yeast as a compensatory response to the loss of the RP copies [4,8]. Cells prefer to duplicate chromosomes containing the remaining RP copies to restore balance, enhancing their fitness despite the aneuploidy.

Discussion; page 23, lines 451-456:
In addition, we found that transcription of *RPS7A* is also under the feedback regulation by Rps7Ap, suggesting that maintaining total level of Rps7p/eS7 is important for yeast physiology. Despite of such regulatory mechanism, deletion of one of RP paralog pairs may lead pivotal effects on growth competition among yeast cells, which sometimes result in survival of mutants with drastic genomic alterations, such as aneuploidy [4, 8].

2. Line 73. What does "delicate to" mean? I don't understand.

We intended to mean that expression levels of *RPS7*-paralog mRNAs are sensitive to modification on their 3'-UTR. To clarify the meaning, we changed the wording as follows.

Page 5, line 82-83:
Overall, yeast has a different buffering system for Rps7p production between the two *RPS7*-paralog mRNAs that are sensitive to a variation on their 3'-UTRs.

3. Line 90 "gene region with an amplified" -> "gene region was replaced with an amplified"

Thank you for pointing out. We revised the corresponding part as below.

Page 6, lines 101-102:
…the disrupted *RPS7B* gene region was replaced with an amplified…

4. Line 192 "phenotype appeared" -> "phenotype that appeared"

Thank you for pointing out. We revised the corresponding sentence as below.

Page 13, lines 229-230:
This growth phenotype that appeared in the *tl(caa)Δint* RPS7A-HA::CgHIS3*…

5. Line 214 to 215 is confusing. Perhaps try, "Unlike tl(caa)deltaint, neither rps7b-101 nor*

rps7b-102 conferred any growth defect at 30C on YPD.

Thank you for your kind suggestion. According to your advice, we revised the sentence as below.

Page 15, lines 262-264:
Unlike *tl(caa)Δint**, neither *rps7b-101* nor *rps7b-102* alone conferred any growth defect at 30°C on YPD (Fig 3B).

6. Line 273 "unchanged" -> "did not change"

Thank you again. We rephrased as below.

Page 19, line 349:
…the *RPS7A* promoter (*rps7a-mut*) did not changed the reporter expression…

7. Line 304, is that a period after strain? should be comma?

Yes, as you suggest, at page 20, line 391 in the revised manuscript with track changes, "strain, and repeated the reporter assay." However, in the revised manuscript, we have updated the sentence as follows.

Page 20, lines 390-392:
Since Fhl1p and/or Rap1p binding sites are crucial for *RPS7*-paralog specific response (Fig 5B), we generated an *fhl1*Δ strain and repeated the reporter assay to analyze whether Fhl1p acts on *RPS7* paralog expression.

8. Lines 325-326 are confusing.

To clarify the meaning, we revised the corresponding sentence as follows.

Page 22, lines 435-437:
Since the production of FLAG-tagged Rps7Bp was well correlated with its mRNA level (Fig 4B–4D), the critical regulatory step in Rps7Bp expression driven by *RPS7A* products seems to rely on mRNA abundance.

9. Line 360 "supposed" -> "expected"

Thank you for your advice. We rephrased the corresponding term as follows.

Page 25, lines 497-498:
expected to coevolve with mRNA properties, …

10. Line 390 "dependet" -> "dependent"

Thank you for pointing out. We corrected the wording as follows.

Page 27, line 544:
…these elements to Rps7Ap-dependent …

11. Line 399 " preference for RPS7Bp". The paper referenced shows a preference for RPS7A, not B in higher protein level.

Thank you for your comment. As you mentioned, the reference (Ghulam *et al.,* 2020, *Nucleic Acids Res.*, 48, 1954-68) shows protein production level of Rps7Ap is higher than that of Rps7Bp. However, they also reveal that ribosomes isolated from polysomes contain more Rps7Bp than Rps7Ap, suggesting that actively translating ribosomes preferentially associate with Rps7Bp over Rps7Ap (Figure 3B and Supplemental Table S5).

12. Line 406 "demonstrates" -> "suggests". demonstrates is a bit too strong.

Based on your comment, we rephrased the wording.

Page 28, line 569:
Our substantiated evidence suggests that the yeast harbors a surveillance system…

13. Line 407 "mRNA level" -> the surveillance or feedback is probably on the protein level or in response to the amount of assembled ribosomes. I don't think there's any evidence that the actual mRNA levels is directly sensed (again why I think it's not "demonstrated"; see comment 12).

Thank you for your opinion. As you pointed out, we do not have solid evidence showing that

cells sense mRNA levels. We added the following description to clarify this point.

Page 28, lines 573-574:
It is important to note that, in many paralogous RP genes, the surveillance or feedback typically occurs through protein level or in response to the amounts of assembled ribosomes.

14. RPS7A mRNA (probably protein, not mRNA). Most likely mechanism is a problem in ribosome assembly, with ribosomes getting stuck in particular stages of assembly. I wonder if there are feedback mechanisms that turn on expression of different genes when different subunits are not assembled well.

We appreciated your thoughtful advice. Based on your comment, we added the description as follows.

Page 28, lines 574-578:
It is also possible that feedback mechanism exists to regulate the expression of different genes when specific subunits are not properly assembled, particularly if ribosomes become stalled at certain stages of translation due to improper expression of paralogous RPs. Comprehensive studies that include both mRNA and protein levels should be considered in future research.
