# Supplementary material for: Beyond the ORF: Paralog-specific regulation of RPS7/eS7 mRNAs via 3’-UTRs and promoter sequences
Source: PLoS One. 2025 May 30;20(5):e0324525. doi: 10.1371/journal.pone.0324525 (PMC12124516; doi:10.1371/journal.pone.0324525)
Supplement: Attachment — Submitted filename: Response to Reviewer.pdf [file pone.0324525.s008.pdf]

Point-to-point answers

5. Review Comments to the Author

Reviewer #3: The authors' revision is much improved. I only have one request. As I mentioned in the first review, it is technically possible that the increase in RPS7B RNA levels that occurs upon deletion of RPS7A could reflect duplication of the chromosome on which RPS7B is found (aneuploidy). The authors should explicitly acknowledge that in the discussion. Currently, they only vaguely hint at the possibility.

Thank you for your comment. We revised the relevant section of the manuscript as follows.

Page 23, line 366:
…deletion of one of RP paralog pairs can lead pivotal effects on growth

Page 23, lines 368-370:
Therefore, in the case of Rps7p regulation, the increase in *RPS7B* mRNA levels that observed in *rps7A*Δ is possibly due to aneuploidy, specifically a duplication of the chromosome that carries *RPS7B*.
